# Organized and Calcified Testicular Hematoma Mimicking a Neoplasm: A Case Report

**DOI:** 10.1002/iju5.70289

**Published:** 2026-10-28

**Authors:** Ahmed Nasir Hanifi, Ahmadullah Hakimi, Murtaza Haidary, Muhammad Ishaque Faizee, Ahmad Zia Gauhary, Mehmood Shah Rahimi, Haider Ali Malakzai, Ahmed Masih Haidary, Mujtaba Hadiari

**Affiliations:** ^1^ Human Medical Laboratories (HML) and Research Center Kabul Afghanistan; ^2^ Medical Research and Technology Center Razi University Kabul Afghanistan; ^3^ Pathology Department Aliabad Teaching Hospital Kabul Afghanistan; ^4^ Department of Pathology and Clinical Laboratory French Medical Institute for Mothers and Children (FMIC) Kabul Afghanistan

**Keywords:** calcified testicular lesion, organized hematoma, testicular hematoma, testicular mass, testicular neoplasm

## Abstract

**Background:**

Calcified intratesticular masses may mimic malignancy. Organized testicular hematomas are rare and may develop fibrosis and dystrophic calcification. Differential diagnoses include regressed (“burned‐out”) germ cell tumors, cavernous hemangiomas, and paratesticular fibrous pseudotumors (PFP).

**Case Presentation:**

A 60‐year‐old man presented with progressive right testicular swelling and dull aching pain. Imaging revealed a 5 × 4 × 4 cm solid, focally calcified intratesticular mass suspicious for neoplasia. Radical inguinal orchiectomy was performed. Histopathological examination revealed dense fibrocollagenous tissue, dystrophic calcification, organized hemorrhage, hemosiderin deposition, cholesterol clefts, hyalinization, and chronic inflammation, without evidence of malignancy.

**Conclusion:**

This case highlights a rare organized, calcified testicular hematoma mimicking neoplasia. Histopathological examination is essential for definitive diagnosis. Organized hematoma should be considered when evaluating calcified intratesticular masses with chronic hemorrhagic and fibrotic changes. Regressed germ cell tumors and cavernous hemangiomas are important alternatives, whereas PFP should be considered when paratesticular origin is uncertain.

## Keynote Message

An organized, calcified testicular hematoma is a rare benign condition that can closely mimic testicular cancer on clinical examination and imaging. This case highlights the importance of considering chronic organized hemorrhage in the differential diagnosis of calcified intratesticular masses, even when there is no history of trauma. Histopathological examination remains essential for establishing the diagnosis and distinguishing benign lesions from malignancy. Recognizing this rare presentation may improve diagnostic awareness, support individualized clinical decision‐making, and help avoid unnecessary radical surgery in selected patients when malignancy can be confidently excluded.

## 1 Introduction

Solid intratesticular masses are generally treated as potentially malignant, particularly because germ cell tumors account for most testicular malignancies [1, 2]. Scrotal Doppler ultrasonography is the primary imaging method and helps characterize lesion morphology and vascularity [3]. However, benign and nonneoplastic lesions can overlap radiologically with tumors, creating diagnostic uncertainty [4]. Calcification may occur in neoplasms, including large‐cell calcifying Sertoli cell tumors [5], and benign vascular lesions such as testicular hemangiomas can also mimic malignancy [6, 7]. Hemorrhage and necrosis may further complicate interpretation [4].

An organized hematoma is a rare nonneoplastic cause of an intratesticular mass. Chronic hemorrhage may organize and develop thrombus, fibrosis, hyalinization, and dystrophic calcification, producing a firm lesion that resembles a tumor. The differential diagnosis includes a regressed (“burned‐out”) germ cell tumor, which may leave fibrosis, calcification, hemosiderin, and inflammation without viable tumor cells [7]. Paratesticular fibrous pseudotumor (PFP) may also show fibrosis and calcification, but usually arises from the tunica vaginalis or other paratesticular structures rather than from testicular parenchyma [8]. We report a calcified testicular mass diagnosed as an organized hematoma mimicking a neoplasm.

## 2 Case Presentation

A 60‐year‐old man presented with progressive right testicular swelling accompanied by dull, aching pain. He denied fever, weight loss, night sweats, urinary symptoms, or previous trauma. His medical and surgical histories were unremarkable. Physical examination revealed an enlarged, firm‐to‐hard, tender right testis. Transillumination was negative, and no inguinal lymphadenopathy was detected.

Scrotal ultrasonography revealed a heterogeneous solid intratesticular lesion measuring approximately 5.07 × 4.02 cm in the longitudinal plane and 5.01 × 3.89 cm in the transverse plane. The lesion contained prominent echogenic foci with posterior acoustic shadowing, suggestive of coarse calcification (Figure 1A,B,D). Color Doppler imaging demonstrated vascular signals predominantly around the periphery of the lesion, with scattered internal flow (Figure 1C). Serum tumor markers were within the reported reference ranges: alpha‐fetoprotein (AFP), 5.2 ng/mL (reference, < 8.4 ng/mL); beta‐human chorionic gonadotropin (β‐hCG), 1.0 mIU/mL (reference, < 5 mIU/mL); and lactate dehydrogenase (LDH), 180 U/L (reference, 122–222 U/L).

**FIGURE 1:**
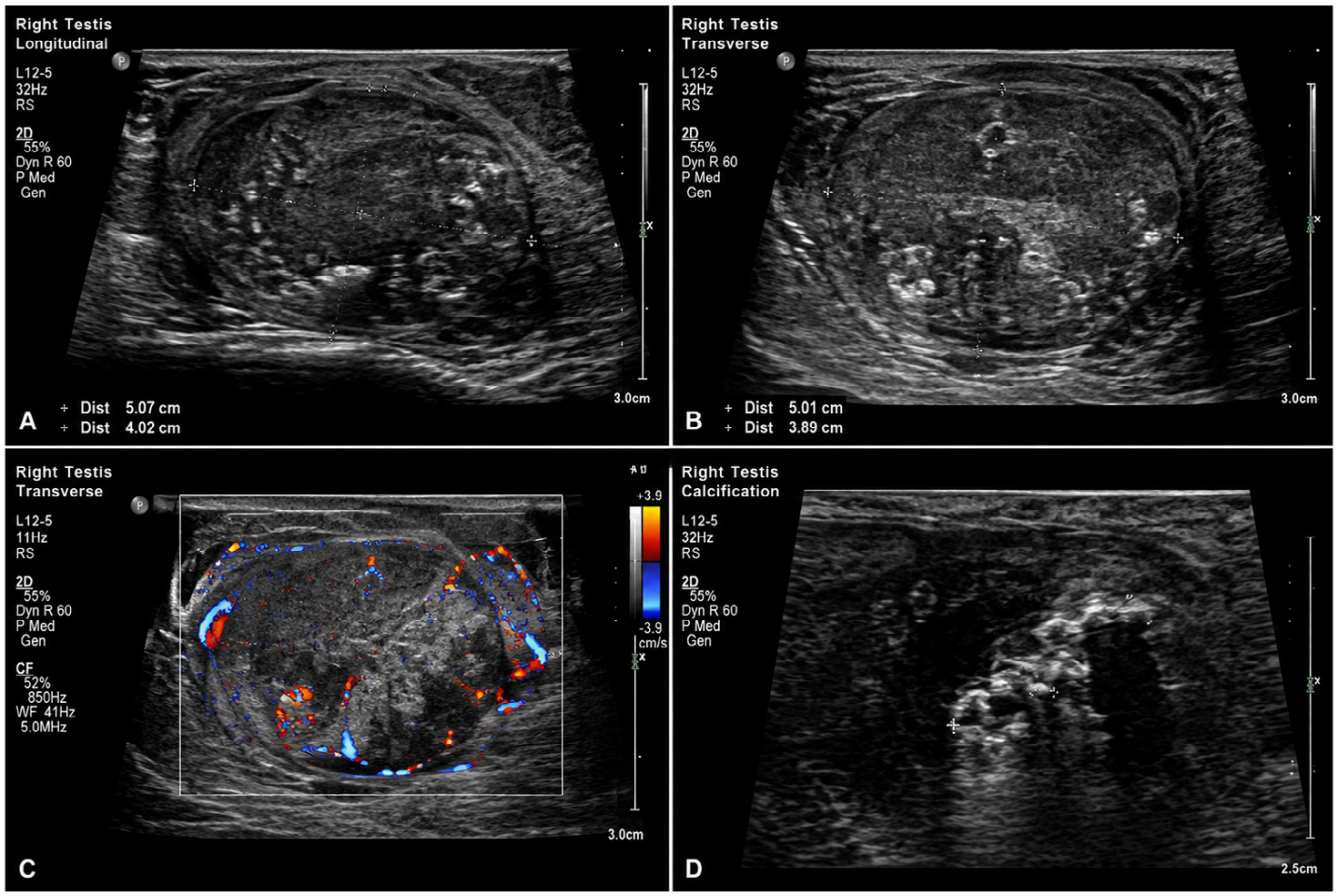
Ultrasonographic findings of the right testicular mass. (A) Longitudinal ultrasound image showing a heterogeneous intratesticular lesion measuring approximately 5.07 × 4.02 cm. (B) Transverse ultrasound image showing the lesion measuring approximately 5.01 × 3.89 cm. (C) Color Doppler image demonstrating vascular signals predominantly at the lesion periphery, with scattered internal flow. (D) Focused ultrasound image showing prominent echogenic foci with posterior acoustic shadowing, consistent with coarse calcification.

Because the lesion was solid and intratesticular and malignancy could not be excluded, radical inguinal orchiectomy was performed. The excised testis measured 9 × 6 × 4.5 cm, and the spermatic cord measured 2 cm in length. Gross examination of the specimen revealed a light‐brown, focally calcified mass measuring 5 × 4 × 4 cm (Figure 2).

**FIGURE 2:**
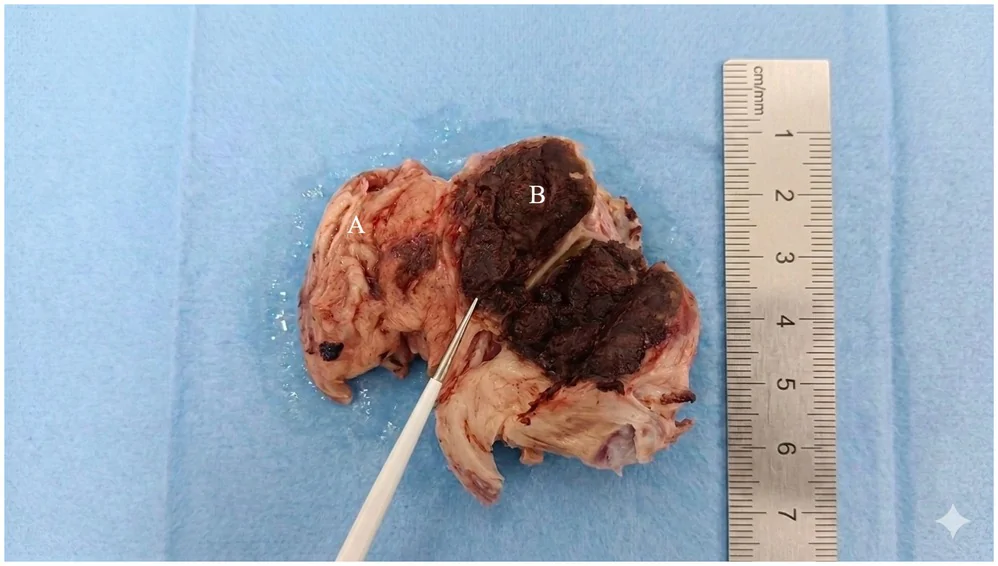
Gross appearance of the orchiectomy specimen showing surrounding fibrotic and paratesticular tissue (A) and an organized hematoma (B), appearing dark red to brown and measuring 5 × 4 × 4 cm.

Histopathological examination demonstrated peripheral basophilic material suggestive of dystrophic calcification (Figure 3A), surrounded by dense, hyalinized fibrocollagenous tissue consistent with chronic fibrosis and organization (Figure 3B). A separate tissue fragment was also present (Figure 3C). Overall, the histopathological findings supported a chronic organized fibrotic process compatible with an organized testicular hematoma. No viable neoplastic proliferation was identified in the examined sections. Considering the clinical presentation, imaging findings, gross appearance, and histopathological features, the final diagnosis was an organized, calcified right testicular hematoma, with no malignancy identified.

**FIGURE 3:**
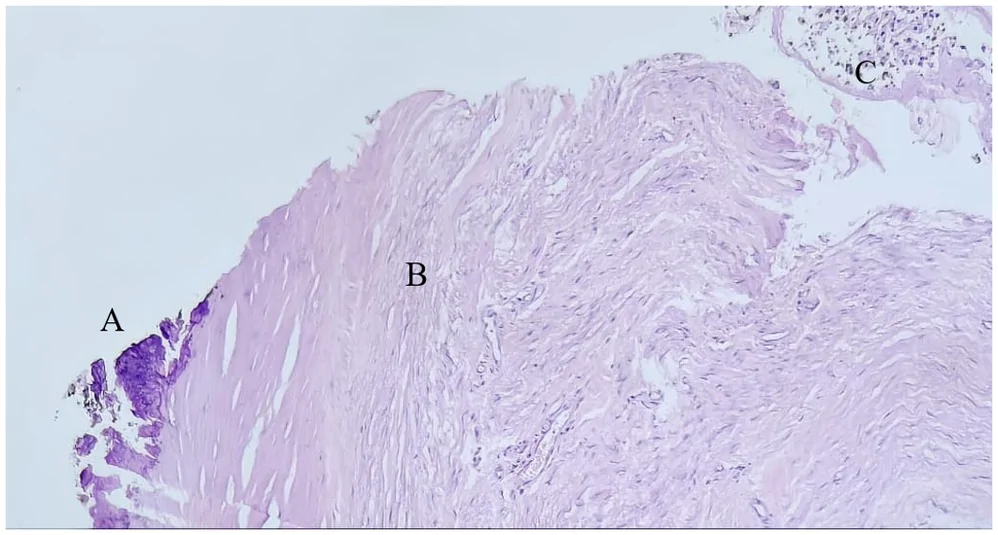
Histopathological features of the organized testicular hematoma (H&E, 10× magnification), showing peripheral basophilic material suggestive of dystrophic calcification (A), dense hyalinized fibrocollagenous tissue (B), and residual seminiferous tubules in the adjacent testicular parenchyma (C).

## 3 Discussion

This case describes an unusual testicular neoplasm mimic in a 60‐year‐old man presenting with progressive swelling, pain, a firm intratesticular mass, and extensive calcification. These findings raised concern for malignancy, particularly because testicular tumors in older men include diffuse large B‐cell lymphoma and seminoma [9]. The 5 × 4 × 4 cm solid lesion demonstrated features that could not reliably distinguish malignancy from chronic hemorrhage. Although calcification occurs in tumors such as large‐cell calcifying Sertoli cell tumors [2], intratumoral bleeding may also produce heterogeneous appearances [1]. Therefore, the imaging features could not reliably distinguish a calcified tumor from a chronic hemorrhagic lesion.

Cavernous hemangioma is another important differential diagnosis because it may calcify and mimic malignancy; dilated, blood‐filled vascular spaces distinguish it histologically [7]. In this case, examination revealed dense fibrocollagenous tissue, organized hemorrhage, hemosiderin deposition, cholesterol clefts, hyalinization, chronic inflammation, macrophages, and dystrophic calcification. These findings supported an organized chronic hematoma. Previous reports describe similar hematomas mimicking testicular neoplasms [10]. Hemosiderin indicates previous bleeding, whereas fibrosis, hyalinization, and thrombus organization reflect chronic lesion development [11]. Progressive organization of the hemorrhagic material and surrounding fibrosis may contribute to the development of a solid, mass‐like lesion. Although extensive sampling revealed no viable malignancy and spermatogenesis was preserved in adjacent seminiferous tubules, a regressed germ cell tumor remains a consideration. Such tumors may leave fibrosis, calcification, hyalinization, and inflammation without viable neoplastic cells [12, 13]. Because the absence of germ cell neoplasia in situ (GCNIS) was not specifically documented, a completely regressed neoplasm cannot be definitively excluded.

The discrepancy between the clinical and radiological findings and the final pathological diagnosis highlights the diagnostic importance of this case. Chronic organized hemorrhage can develop fibrosis and dystrophic calcification, forming a firm mass that resembles a primary testicular tumor. PFP is another benign mimic, although its usual origin in the tunica vaginalis or other paratesticular structures helps distinguish it anatomically [7]. The cause of hemorrhage remains uncertain. Potential causes include trauma, torsion, infection, coagulation disorders, neoplasms, vasculitis, and anticoagulant therapy [14, 15]. No trauma or previous surgery was reported, and the underlying cause could not be established. Chronic expanding hematomas may involve inflammation, fragile neovascularization, and recurrent bleeding [11].

Radical inguinal orchiectomy was performed because malignancy could not be excluded preoperatively. Testis‐sparing surgery and frozen‐section examination may be appropriate for selected indeterminate lesions, whereas orchiectomy remains important when malignancy is strongly suspected [16]. Organized hematoma should be considered in the differential diagnosis of calcified intratesticular masses. The principal limitation is the unidentified cause of hemorrhage and the inability to definitively exclude a completely regressed neoplasm. Histopathological examination remains essential for definitive diagnosis.

## 4 Conclusion

An organized and calcified testicular hematoma is a rare benign lesion that can closely mimic testicular malignancy. In this patient, organized hemorrhage and dystrophic calcification supported the diagnosis, with no viable malignancy identified. Organized hematoma should be included in the differential diagnosis of calcified intratesticular masses. Regressed germ cell tumor and cavernous hemangioma should also be considered, whereas PFP is relevant when paratesticular origin is possible. Histopathology remains essential for definitive diagnosis, and a regressed neoplasm cannot be completely excluded solely because viable tumor cells are absent.

## Funding

The authors have nothing to report.

## Ethics Statement

The research protocol was reviewed and approved by the Institutional Review Board of Khatam Al‐Nabieen University, Kabul, Afghanistan (Approval No. KNU.AFG.2026‐04‐1).

## Consent

Written informed consent was obtained from the patient for publication of this case report and any accompanying clinical images.

## Conflicts of Interest

The authors declare no conflicts of interest.

## Data Availability

The data that support the findings of this study are available from the corresponding author upon reasonable request.
